# AC-YVAD-CMK Inhibits Pyroptosis and Improves Functional Outcome after Intracerebral Hemorrhage

**DOI:** 10.1155/2018/3706047

**Published:** 2018-10-16

**Authors:** Xiao Lin, Haotuo Ye, Felix Siaw-Debrah, Sishi Pan, Zibin He, Haoqi Ni, Zhu Xu, Kunlin Jin, Qichuan Zhuge, Lijie Huang

**Affiliations:** ^1^Department of Neurosurgery, The First Affiliated Hospital of Wenzhou Medical University, Wenzhou 325000, China; ^2^Zhejiang Provincial Key Laboratory of Aging and Neurological Disorder Research, The First Affiliated Hospital of Wenzhou Medical University, Wenzhou 325000, China

## Abstract

Intracerebral hemorrhage (ICH) refers to bleeding in the brain and is associated with the release of large amount of inflammasomes, and the activation of different cell death pathways. These cell death pathways lead to removal of inactivated and damaged cells and also result in neuronal cell damage. Pyroptosis is a newly discovered cell death pathway that has gained attention in recent years. This pathway mainly depends on activation of caspase-1-mediated cascades to cause cell death. We tested a well-known selective inhibitor of caspase-1, AC-YVAD-CMK, which has previously been found to have neuroprotective effects in ICH mice model, to ascertain its effects on the activation of inflammasomes mediated pyroptosis. Our results showed that AC-YVAD-CMK could reduce caspase-1 activation and inhibit IL-1*β* production and maturation, but has no effect on NLRP3 expression, an upstream inflammatory complex. AC-YVAD-CMK administration also resulted in reduction in M1-type microglia polarization around the hematoma, while increasing the number of M2-type cells. Furthermore, AC-YVAD-CMK treated mice showed some recovery of neurological function after hemorrhage especially at the hyperacute and subacute stage resulting in some degree of limb movement. In conclusion, we are of the view that AC-YVAD-CMK could inhibit pyroptosis, decrease the secretion or activation of inflammatory factors, and affect the polarization of microglia resulting in improvement of neurological function after ICH.

## 1. Introduction

Intracerebral hemorrhage (ICH) is a significant clinical emergency, which affects several million people worldwide [1]. ICH results in high mortality and morbidity and also causes serious disability and dependency throughout the world [2]. Over the years, many scientific researchers have focused on exploring potential mechanisms that may influence positively the outcome of ICH. The outcome of these studies has revealed targeting and attenuating increase release of proinflammatory cytokines, apoptosis, autophagy, and other cell death mechanisms around the hematoma region as major factors that influence the outcome [3, 4]. Recently, pyroptosis is another important cell death mechanism that has received much attention. The pyroptotic pathway consists of inflammasomes, which include apoptosis-associated speck-like proteins, caspase-recruiting domain (ASC), adapter protein, and caspase-1, which is an inflammatory cysteine-aspartic proteinase [5]. This pathway, unlike apoptosis and autophagy, is mainly mediated by caspase-1 signaling pathway and is a cysteine aspartate protease-mediated programmed self-death [6, 7]. Activation of pyroptotic pathway not only activates inflammatory cytokines, but also induces cell death after proinflammatory intracellular content is released [5]. NLR protein-3 (NLRP3) inflammasome is a classic example of an inflammatory body. It consists of a variety of proteins including NLRP3, ASC, and caspase-1, which, when activated, breaks down caspase-1 into two subunits, p20 and p10. These subunits further activate the secretion or release of IL-1*β* and IL-18 [8]. Many experiments have confirmed caspase-1-mediated pyroptosis to be involved in various inflammatory processes [9] as well as many neurologically related diseases [10, 11]. It is therefore not surprising that NLRP3 inflammasome dependent inflammatory response has been seen as a therapeutic target after intracerebral hemorrhage [12].

Microglia cells are considered resident immune cells of the central nervous system (CNS). In the mature brain, microglia cells maintain homeostasis and exist in a resting ramified state [13]. The progression and resolution of many CNS diseases are therefore dependent on the participation of activated microglia. While some microglial processes may be beneficial, microglia have also been shown to play a role in the secondary injury that occurs after ICH [14], as well as in other pathological processes of various neurological diseases, such as Alzheimer's disease, Parkinson's disease, and stroke [15–17]. Various studies have indicated that activated microglia have two main types, that is, M1 and M2. M1 is the classical pathway activation type, mainly secreting proinflammatory cytokines such as tumor necrosis factor-alpha (TNF-*α*), iNOS, and chemokine 2 and other molecules. The M2-type is a selective activation type and specifically expresses molecules such as CD206, CD68, and Arg-1 and releases interleukin 10(IL-10), transforming growth factor beta (TGF-*β*) and other molecules, which mainly plays a protective role against brain damage [13]. Although many studies have indicated that M1 microglia are activated during ICH episode leading to the progress of inflammatory reaction, others have also reported activation of M2 cell to protect brain from damage [18]. A clearer understanding into microglia polarization is still needed especially since little is known about the association between microglia and pyroptosis.

AC-TYR-VAL-ALA-ASP-CMK (AC-YVAD-CMK) is a well-known selective inhibitor of caspase-1 proven to have therapeutic effect on ischemic stroke [19], but the specific mechanism of action still remains unknown. Considering this known fact, we hypothesize that AC-YVAD-CMK could be used to treat collagenase induced ICH in mice model. We injected the drug into the mice brain and analyzed brain tissue for both inflammatory and anti-inflammatory protein expression and, at the same time, observed changes in mice behavior to explore the efficacy and potential mechanisms of drug.

## 2. Materials and Methods

### 2.1. Animals and Groups

All clean grade male C57BL/6 mice with weight of 24-28g were purchased from the Shanghai Slaccas Experimental Animal Limited Liability Company (Shanghai, China). In a temperature-controlled room, mice were given food, water, and room light simulating normal day and night changes. The experiment was approved by the Animal Experimental Center of Wenzhou Medical University and was under its supervision.

Fifty-six mice were randomized into 4 different groups using computer-generated random numbers [20]: a normal group (normal, n = 14), a sham operation group (sham, n = 14), an intracerebral hemorrhage treated with vehicle group (ICH, n = 14), and an intracerebral hemorrhage treated with AC-YVAD-CMK group (ICH+T, n = 14). All the experimental proceedings were performed in a blinded manner.

### 2.2. ICH Model

The collagenase injection method was used to induce intracerebral hemorrhage in experimental mice [21]. The mice were intraperitoneally anesthetized with 4% hydrochlorate (0.01 ml/g; Solarbio, Beijing, China) and fixed on the stereotactic head frame (Kopf Instruments, Tujunga, CA). Using a mini electric blanket to keep the rectal temperature at 37°C ± 0.5°C, the mice heads were shaved and a 1 cm long incision at the middle of the scalp was made, to expose mice skull. A 1 mm hole was drilled using a microdrill at 0.2 mm anterior to the bregma and 2.0 mm right to skull midline. Using a stereotactic 26-gauge needle inserted at about 3.7 mm deep in the right striatum, we injected collagenase VII-S (Sigma, C2399) into the brain using a microinfusion pump (KDS legato) at a concentration of 0.075U collagenase in 0.5 *μ*l saline. The injection speed was constantly maintained at 2 *μ*l/min by the microinfusion pump. Infusion needles were, however, left in place for 10 minutes and then removed at a rate of 1 mm/min after infusion was over. The sham group was, however, subjected to all surgical procedures but without collagenase infusion. Incision was sutured, and the mouse returned to its cage for recovery. In total, 3 experimental mice were recorded dead. Two mice died after injection of anesthesia (1 in each group, sham group and ICH+T group), with one dying from excessive injection of collagenase due to error in calibration of syringe pump. Mice were anesthetized and sacrificed after 24 h of ICH induction, and brain samples harvested. Samples obtained for the purpose of immunohistochemistry were stored in paraformaldehyde (40g/L) at 4°C, whilst those for western blot were stored in liquid nitrogen after harvesting and later transferred to -80°C storage (not more than 24 h before protein isolation).

### 2.3. Drug Injections

As mentioned earlier [22], AC-YVAD-CMK was injected by intraventricular route, 15 minutes prior to collagenase injection. Preparation of AC-YVAD-CMK was done by dissolving solid AC-YVAD-CMK in DMSO and diluted further with phosphate buffer (PBS, final concentration DMSO = 0.1%) to a concentration of 400 ng/1 *μ*L for every mouse. The drug was injected into the ventricle at a site 1 mm left and 2.5 mm ventral to the bregma. Vehicle group (ICH group) was injected with equal volume of vehicle (0.1%DMSO) before ICH. Sham group, however, had only needle insertion without any treatment protocol.

### 2.4. Western Blot Analysis

Western blot was used to determine the amount of tissue protein expression as previously described [20]. A 2 mm thick coronal section of the ipsilateral hemisphere was collected from each group of specimens. Tissues were put into ice-cold radio-immunoprecipitation assay lysis buffer (RIPA) with phenylmethanesulfonyl fluoride (PMSF) (RIPA: PMSF=100:1) for 30 min and then crushed with tissue homogenizer. The homogenate was then transferred to a microtube and centrifuged at 12000 rad/min for 20 minutes in a precooled centrifuge at 4°C. The BCA kit (Beyotime) was used to test the protein concentration. The protein of interest was separated by SDS-PAGE and then transferred to PVDF membrane, followed by blocking with 5% milk for 2 hours, and then incubated overnight with primary antibodies, that is, caspase-1 (1:1000, ab1872, abcam, RRID:AB_302644), IL-1*β* (1:1000, abcam, RRID:AB_308765), cleaved-caspase-1 (1:500, ab1872, abcam, RRID:AB_302644), cleaved- IL1 beta (1:500, abcam, RRID:AB_308765), and GAPDH (1:1000, 5174S, Cell Signaling Technology, AB_10622025) at 4°C.After 24 hours; membranes were further incubated with secondary antibodies (1:5000) at room temperature for 1 hour. Band results were quantified using Image Lab software (Bio-Rad), and the target protein signals intensities were compared with *β*-actin or GAPDH intensity.

### 2.5. Brain Collection and Immunohistochemistry

Mice were anesthetized and sacrificed with 4% paraformaldehyde via transcardial perfusion. Brain tissues were quickly removed and fixed in 4% paraformaldehyde at 4°C for two days. After two days, tissues were fixed in 30% sucrose until they sank. The brain was embedded in O.C.T. Compound (SAKURA,4583) fixed on the frozen microtome and sliced to a thickness of 8 um. To determine the activation and polarization of microglia, immunofluorescent labeling using Iba1 labeled microglial, iNOS-labeled microglial M1, and CD206-labeled microglial M2 was done. As mentioned earlier, sections were soaked in paraformaldehyde and then rinsed with PBS. This was then blocked with 10% NDS (Normal Donkey Serum, dissolved in PBS) + 0.4% Triton-X for 1 hour, followed by incubation in antiserum to Iba1 (1:800, ab5076, Abcam, AB_2224402) and either iNOS (M1 marker; 1:300, ab15323, Abcam, AB_301857) or CD206 (M2 marker; 1:300, ab8918, Abcam, AB_306860) in 10% NDS + 0.4% Triton-X at 4°C for 12-16 hours. After 24 hr, sections were incubated with appropriate secondary antibodies conjugated to AlexaFluor-594 (Invitrogen, 1:300) and 488 (Invitrogen, 1:300). They were rinsed extensively in PBS +5% NDS for about 1 h and covered with DAPI (Vector Laboratories) for about 5 min. Sections were then examined using a scanning-fluorescence microscope (Leica Microsystems), at 40× objective, while image J software was used to determine colabeling between Iba1 with M1 or M2 markers. Statistical analysis from 6 randomly selected stained sections of brain tissue was done. Similarly, six digital micrographs at the marginal zone of the hematoma of each section were randomly taken. The numbers of double positive cells were selected, and the average of the slices of each brain was calculated (n = 3 for each group).

### 2.6. Neurologic Deficit

Each experimenter was blinded to all operational mice accessed for neurologic deficits with modified neurological severity score (mNSS) 7d after ICH. The tests included limb symmetry, exercise capability, balance ability, circling behavior, reflexes, and abnormal movements, with a maximum deficit score of 18 [23]. Before ICH, all mice were trained for three days.

### 2.7. Rotarod Test

Motor impairment was accessed using the rotarod test. The rotarod test was done as described previously [24]. Mice were trained for five consecutive days before ICH. The test, which lasted for 5 minutes, involves increasing the speed from 5 revolutions per minute (rpm) to 40 rpm, recording the time till the animal was felled from the drum (in seconds). After training, if the experimental animal could not last for about 250s on the instrument, they were excluded from the experiment. Each session included three training sessions, with each lasting about 300 seconds. The next training is done on an average interval of 20 minutes. The final calculation involves taking the average of the three. In this study, all experimental mice were able to meet the requirements after training.

### 2.8. Statistical Analyses

The statistical analysis of the experimental data was done using SPSS 22.0 software and Prism 5.0. All time related results were expressed as means ± standard deviation (SD). One-way ANOVA was used in western blot and immunohistochemistry. For neurologic deficit and rotarod test, repeated measures ANOVA analysis and two-way ANOVA were used to determine the difference between groups. Results were only considered to be statistically significant at P < 0.05.

## 3. Results

### 3.1. AC-YVAD-CMK Had No Effects on NLRP3 Inflammasome after ICH

**Figure 1:**
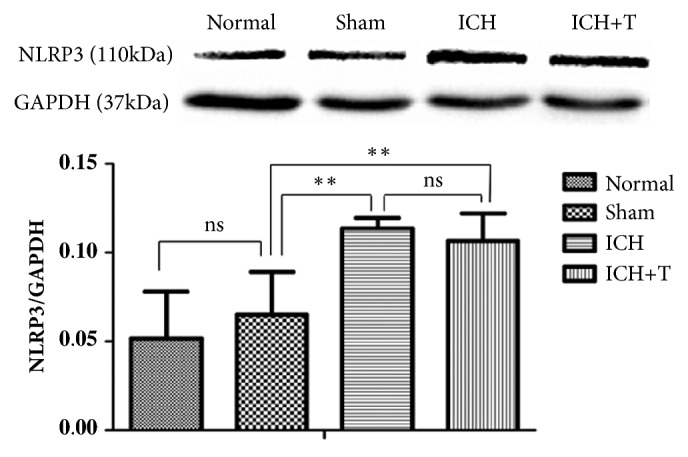
Protein levels were determined by western blotting. NLRP3 levels showed no obvious changes between ICH and ICH+T group, with similar observation between normal and sham group. However, there was significant difference between sham group and both ICH and ICH+T group, (*∗∗*P <0.01, ns=no significance). n=4 for each group. One-way ANOVA.

Western blot analyses showed increased NLRP3 protein expression in ICH group and ICH+T group compared with normal and sham group. However, there was no significant change between ICH group and ICH+T group (Figure 1).

### 3.2. AC-YVAD-CMK Inhibited Caspase-1 Activation and Reduced the Amount of Mature IL-1*β* after ICH

**Figure 2:**
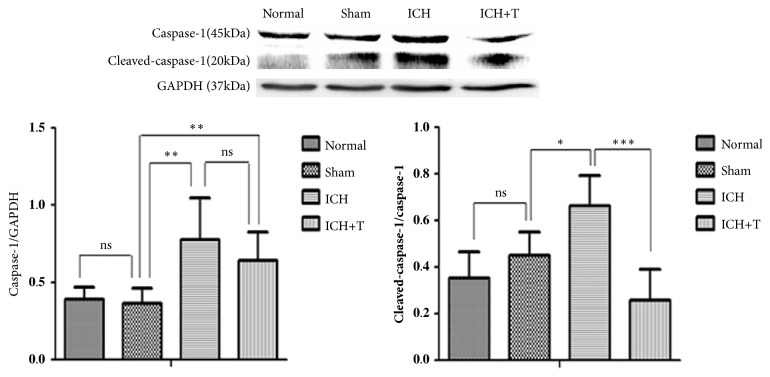
Caspase-1 expression by western blot. Sham group compared with the normal group showed no obvious difference between caspase-1 and cleaved-caspase-1/caspase-1 expression. However, compared with the sham group, both caspase-1 and cleaved-caspase-1/caspase-1 showed significant increase after ICH. ICH+T group also showed lower expression of caspase-1 compared with ICH group (*∗*P <0.05, *∗∗*P<0.01, *∗∗∗*P<0.001, ns=no significance), n=4 for each group. One-way ANOVA.

**Figure 3:**
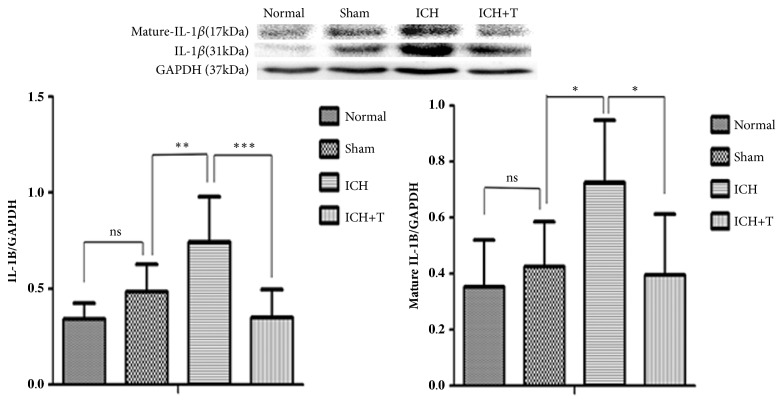
IL-1*β* expression by western blot. There was no significant difference between normal and sham group. Expression of IL-1*β* and mature IL-1*β* increased compared to the sham group after ICH. The ICH+T group showed decrease expression of IL-1*β* and mature IL-1*β* as compared to ICH group (*∗*P <0.05, *∗∗*P<0.01). (*∗∗∗*P <0.001*∗∗*P <0.01, *∗*P<0.05, ns=no significance). n=4 for each group. One-way ANOVA.

Western blot analyses showed that caspase-1 continued to increase 24 hours after ICH in ICH group compared with the sham group. However, activation was significantly inhibited in AC-YVAD-CMK-treated mice compared with untreated mice. The ratio of activated caspase-1 to precursors showed a significant decrease (Figure 2). After 24 hours of ICH an increased expression ofIL-1*β* and mature-IL-1*β* was observed. Pretreatment with AC-YVAD-CMK, however, reduced the production of IL-1*β* and mature-IL-1*β* (Figure 3).

### 3.3. AC-YVAD-CMK Decreased the Activation of M1 Microglial and Increased M2 Microglial Cells

**Figure 4:**
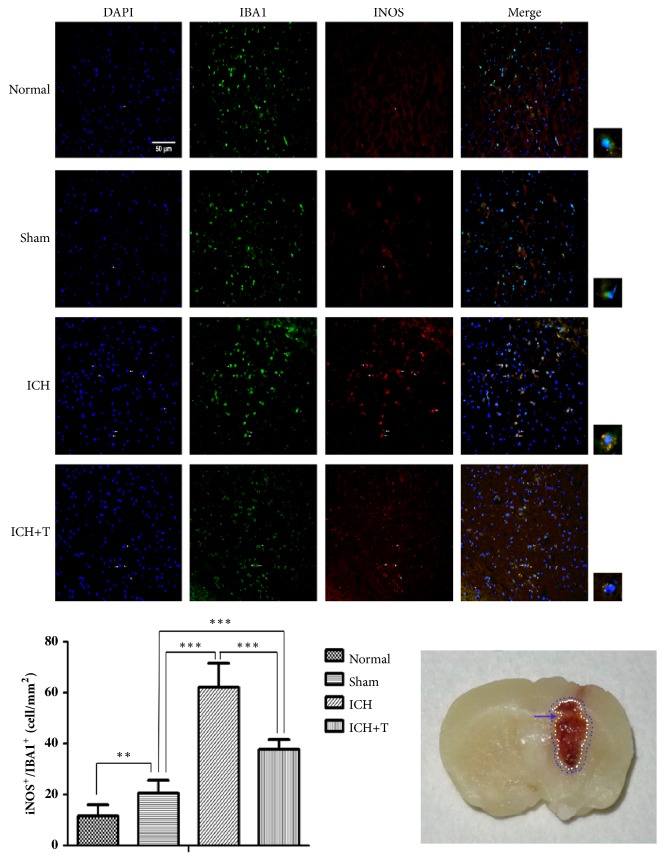
AC-YVAD-CMK decreased iNOS+/Iba1+ cells. ICH group showed increased iNOS+/Iba1+ cells 1 day after ICH as compared to the sham group. ICH+T, however, showed significantly decreased iNOS+/Iba1+ cells 1 day after ICH group compared with ICH group. Arrows indicate iNOS+/Iba1+ cells (*∗∗*P < 0.01,*∗∗∗*P < 0.001, n = 3). ICH+T: treated with AC-YVAD-CMK. ICH: treated with DMSO. The region between the red and blue cycle (blue arrow) indicates the area around hematoma, where images were taken. Scale bar= 50 *μ*m. (The white arrow in immunofluorescence staining picture indicates the stained area; the white square shows the costained cells, which is the enlarged part.) One-way ANOVA.

**Figure 5:**
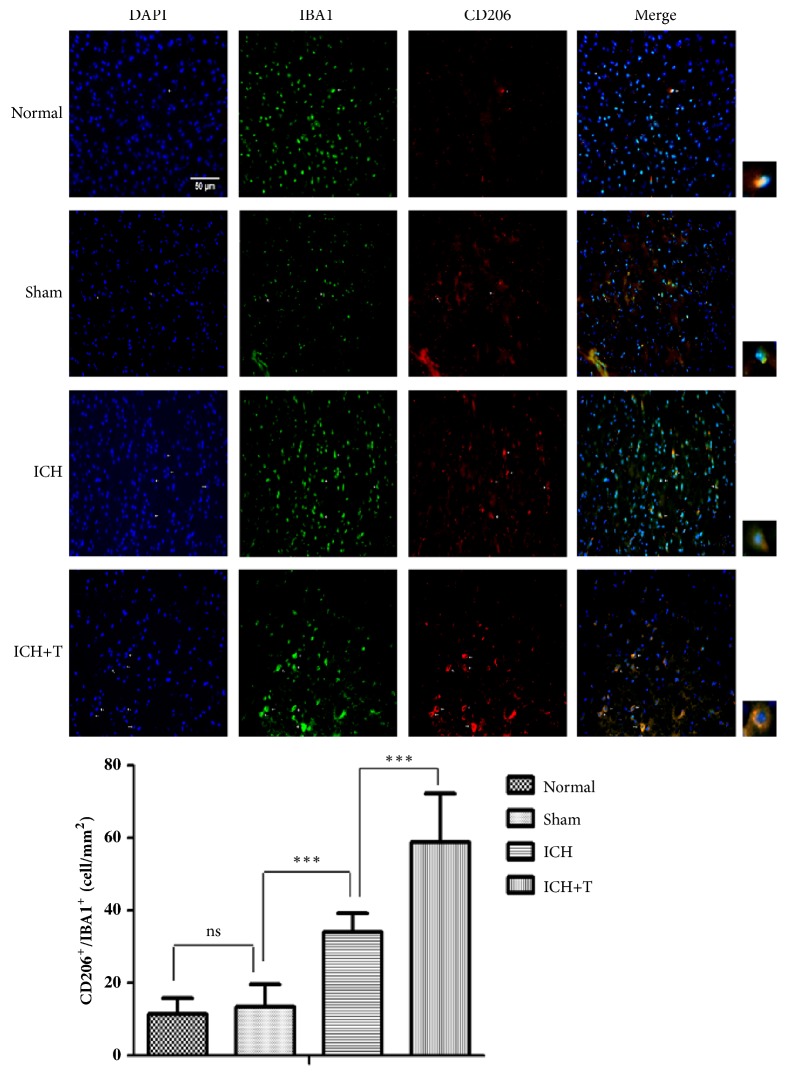
AC-YVAD-CMK increased CD206+/Iba1+ cells. ICH group compared with sham group showed increased CD206+/Iba1+ cells 1 day after ICH. ICH+T group compared with ICH group also demonstrated significant increase in CD206+/Iba1+ cells. Arrows indicate CD206+/Iba1+ cells (*∗∗∗*P < 0.001, n = 3). ICH+T: treated with AC-YVAD-CMK. ICH: treated with DMSO. Scale bar= 50 *μ*m. One-way ANOVA.

To determine the activation state of microglia after ICH, we collected mouse brain slices, and sections were colabeled with Iba1 and either M1 markers (iNOS) or M2 markers (CD206). Confocal analysis showed that Ac-YVAD-CMK could significantly decrease the double positive cell ratio of iNOS+/Iba1+ cells surrounding the hematoma 1 day after ICH (Figure 4). The costaining CD206/Iba1 analyses showed that CD206+/Iba1+ cells increased markedly in the ICH+T group as compared to ICH group (Figure 5). In summary, the results show that pretreatment with Ac-YVAD-CMK can inhibit activation of M1 microglial and can also enhance M2 microglial activation after ICH.

### 3.4. AC-YVAD-CMK Improved Neurological Behavior after ICH

**Figure 6:**
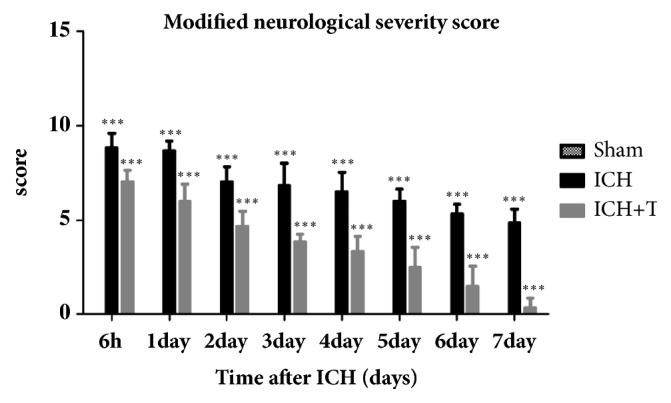
The sham group did not show any neurological dysfunction after surgery. The mNSS score of ICH group was significantly higher than sham group seven days after ICH (*∗∗∗*P<0.001). Pretreatment with Ac-YVAD-CMK improved modified neurological severity score 7 days after ICH. mean ± SD, Two-way ANOVA. The treatment group in comparison with the ICH group showed significant difference, and interaction was obvious between the treatment method and the test time; repeated measure ANOVA; n=6 for each group (*∗∗∗*P<0.001). ICH+T: treated with Ac-YVAD-CMK. ICH: treated with DMSO.

The normal group had 0 score and, as such, were not placed on the figure. The sham group did not show any neurological dysfunction after surgery. An obvious functional deficit was exhibited in ICH group 7d after ICH. ICH group compared with the sham group shows significant functional impairment. The modified neurological severity score (mNSS) assessment showed no effects of vehicle in the vehicle group, while ICH+T group showed significant improvement in neurological function (Figure 6). This difference first appeared in the sixth hour after ICH and then after seven days.

### 3.5. AC-YVAD-CMK Improved the Result of Rotarod Test

**Figure 7:**
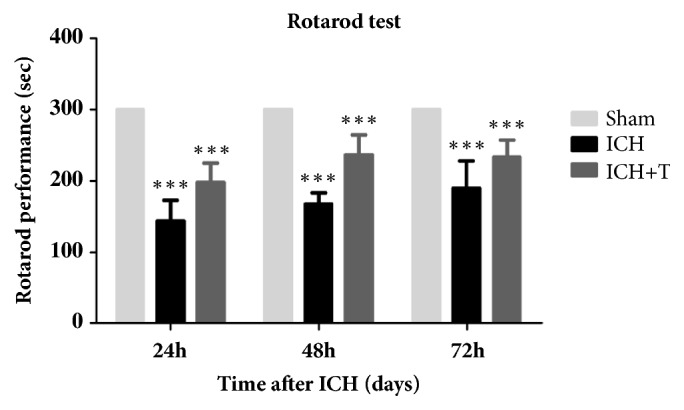
The sham group did not change in rotarod performance after surgery. The duration of the ICH mice decreased significantly compared with the sham group after 72 h of ICH (*∗∗∗* P<0.001). AC-YVAD-CMK treatment improved rotarod performance time after 72 hrs as compared with ICH group (*∗∗∗* P<0.001, *∗∗* P<0.01). mean ± SD, Two-way ANOVA. Significant difference was established between the ICH and treatment group; however, there was no interaction between the treatment method and the test time; repeated measure ANOVA; n=6 for each group. ICH+T: treated with Ac-YVAD-CMK. ICH: treated with DMSO.

As described earlier, different groups used the same acceleration method. The duration of the test results of the normal group mice is greater than 300s; it was not shown in the figure. In the sham group, mice were found not to have neurological deficits in the rotarod test, but ICH mice showed severe neurological deficits following surgery. After treatment with AC-YVAD-CMK, mice showed significant improvement of motor impairment compared with ICH group (Figure 7), n=6 for each group.

## 4. Discussion

During an episode of acute intracerebral hemorrhage, many inflammatory cascades are triggered around the hematoma area leading to the release of cytokines and the breakdown of the blood-brain barrier. These factors together with other inflammatory factors further aggravate the migration of systemic inflammatory cells to the hematoma area [25, 26]. NLRP3 inflammasome is widely studied and accepted to play a crucial role in the inflammatory process in the nervous system, especially after ICH [12].

NLRP3 plays an important role in the initial process of pyroptosis during an ICH episode. This is demonstrated in the elevation of NLRP3 inflammasome expression and activation of caspase-1 after ICH. AC-YVAD-CMK is a well-known specific inhibitor of caspase-1. However, whether AC-YVAD-CMK exerts inhibitory effect on the expression of NLRP3 after ICH is still unknown. In this study, we discovered increased NLRP3 expression after ICH and at the same time, we noticed increased caspase-1 activation, which was probably induced by NLRP3 inflammasome. Administration of AC-YVAD-CMK was seen to attenuate caspase-1 activation. However, there is no evidence suggested effect on the expression of NLRP3. AC-YVAD-CMK administration led to decreased levels of inflammation as well as pyroptosis. Because pyroptotic cell death depends on caspase-1 activation, AC-YVAD-CMK administration may have, therefore, attenuated caspase -1 activation, hence inhibiting caspase-1-mediated pyroptotic cell death.

Our experiment used a suitable dosage of 400 ng of AC-YVAD-CMK according to the results of Hideaki's study for ischemic neuronal damage [19]. This was due to the fact that dosages greater than 800 ng per mice led to the death of mice, whereas the dosages less than 200 ng had no significant effect. We, therefore, used a dosage of 400 ng as our optimum dosage throughout our experiment. In this study, results showed that activated NLRP3 inflammasome, including NLRP3, caspase-1, as well as activated cleaved-caspase-1 (p20) and IL-1*β*, had increased expression in ICH mice. A comparison between the ICH induced group and the control showed increased expression of NLRP3 and caspase-1, and an increased level of circulating IL-1*β*. This was probably due to the triggered activation of NLRP3 inflammasome pathway, which aggravates the release of IL-1*β*. Mice administrated with AC-YVAD-CMK, on the other hand, showed the reverse with significant reduction of caspase-1 expression, downregulating the mature IL-1*β*. These results indicate that NLRP3 inflammasome is most likely activated during an ICH episode leading to NLRP3-induced caspase-1 dependent inflammatory response. Therefore, targeted inhibition of caspase-1 activation or NLRP3 inflammasome activation may serve as a potential new therapeutic target for inhibiting the pyroptosis after ICH.

Microglia, innate immune cells of the central nervous system, occupy a very important position in the occurrence and progression of nervous system diseases. Recent studies have shown that M1-signature (proinflammatory) cytokines significantly rise after ICH. For example, activated nuclear factor-*κ*B (NF-*κ*B) was seen to migrate into the nucleus at 13–48 h after ICH and at the same time, IL-1*β* and tumor necrosis factor (TNF) levels increased within 1 day after ICH, while downregulation of TNF-*α*expression was associated with reduction in hematoma volume [27–29]. Some researchers found that M1 markers such as CD16, CD32, and iNOS are highly expressed on microglia on days 1 and 3 after ICH [30], suggesting that M1 cells' polarization occurs early in the acute phase. M2-type microglia are considered to be protective cells. Induction of microglia M2 polarization is considered to be beneficial against damaged brain from nervous system injury disease like ischemic and spinal cord injury [31, 32]. This study found that IL-4 administration can promote M2 polarization and reduce ischemic lesion volume, and lipopolysaccharide preconditioning improves spinal cord injury by facilitating M2 activation. Similarly, the resolution of hematoma volume was seen to be strongly associated with selective activation of M2 microglia, which promoted phagocytosis [33]. Recent experimental findings have indicated that M1 polarization of microglia will increase after ICH evident by an increase in interleukin 1-beta (IL-1*β*) secretion [34]. Another study found that NLRP3 is mainly concentrated in astrocytes and microglia in the CNS [35, 36]; as such, the production of NLRP3 and IL-1*β* is, therefore, associated with microglia activation to some extent. Our study found that pretreatment of ICH model with AC-YVAD-CMK could inhibit the activation of caspase-1 and reduce the production and maturation of IL-1*β* without affecting NLRP3. Therefore, we hypothesized that AC-YVAD-CMK might have affected the microglia changes around the hematoma. In order to prove whether the microglial cells around the hematoma after ICH were changed, we performed immunofluorescence staining from frozen sections of mouse brain tissue, for which results showed significant microglia activation around the hematoma especially that of M1 microglia. It was shown that M1 microglia increased significantly in the surrounding area after ICH, while M2 microglia only slightly increased. This result is consistent with the findings of other researchers who studied changes in microglia after ICH [37]. The main effect of microglial activation and polarization after ICH is the release of inflammatory cytokines and phagocytic debris [14]. However, the effect after pretreatment with AC-YVAD-CMK resulted in significant decrease in M1 polarization, more prominent after ICH 24 h, whereas the proportion of M2 cells increased. This shows that mice with AC-YVAD-CMK treatment showed signs of microglia polarization from M1 to M2 around the hematoma region favoring the migration of more M2 to this region. M2 microglial cells have been proved to possess a neural repair effect. It can secrete anti-inflammatory cytokines and increase neuroprotective factors in the central nervous system [34]. Therefore, we are of the view that this change in microglia polarization favoring M2 microglia may be the key to improving neurological impairment after ICH. Although the mechanism of this change is still unknown to us, further research is needed to help explain this phenomenon. In addition, changes in the expression of the pyroptotic proteins and the changes in the bound microglia suggest that microglia in the brain had M1-type polarization after ICH, which resulted in a large number of NLRP3 inflammasomes and caspase-1 activation leading to subsequent release of IL-1*β* and pyroptotic response. With treatment of AC-YVAD-CMK, the activation of caspase-1 was blocked, which prevented the release of subsequent inflammatory factors and, at the same time, promoted the transformation of microglia into M2-type, exerting anti-inflammatory effects, leading to improved neurological function.

From our results, we deduced that AC-YVAD-CMK could inhibit pyroptosis by inhibiting the expression and activation of other inflammatory factors and also change the polarization state of microglia. This positive effect was seen in the AC-YVAD-CMK pretreatment group demonstrated by the improvement in neurological function as recorded by mNSS and rotarod test. We discovered that the drug treated group showed significant improvement after ICH mainly due to the attenuation of further injury due to aggravated inflammation. Our assumption is that AC-YVAD-CMK improves nerve function by inhibiting pyroptosis and providing neuroprotection after acute ICH episode. Furthermore, we found that administration of AC-YVAD-CMK not only improved neurological impairment, but also improved limb mobility and fatigue tolerance after ICH. In conclusion, we hypothesize that AC-YVAD-CMK improving neurological function may be related to its inhibition of pyroptosis and promotion of microglial cell polarization to M2-type, so it gives an anti-inflammatory effect. In addition, AC-YVAD-CMK have been shown to reduce brain edema at 24H and 72H after ICH [38]. But whether it can affect the change of cerebral hematoma volume is unknown. We will examine it in subsequent experiments.

Not until recently have many researchers thought that there was no difference between pyroptosis and apoptosis. However, animal studies of intracerebral hemorrhage have discovered the presence of apoptotic cells in brain tissue 6 h after injection of autologous blood, which was accompanied by higher caspase-3 gene expression [39]. In another experiment, apoptosis was seen to be mediated by caspase protease, but with caspase-3 as its key enzyme [31]. Ferroptosis is a newly discovered cell death pathway in recent years. Discovered by Dixon, ferroptosis is characterized by the overwhelming, iron-dependent accumulation of lethal lipid ROS. This requirement for ROS accumulation appears to be universal. In some cell studies, NOX-family enzymes make important contributions to this process [40]. It is well known that there is a large amount of iron accumulation after ICH, so whether ferroptosis is involved in aggravating effect of ICH is a new direction of investigation. Recent studies on ferroptosis have reported that the administration of ferrostatin-1, a known specific inhibitor of ferroptosis, resulted in the prevention of neuronal death after ICH. Ferrostatin-1 was documented to reduce iron deposition induced by hemoglobin in experimental ICH mice, thereby promoting neurologic function recovery, indicating the likely role of ferroptosis in the disease process of ICH [41]. In contrast to earlier mentioned cell death pathways, pyroptosis is mainly dependent on caspase-1 and not caspase-3 pathway, although the specific mechanism remains unclear. At present, there are many studies on the detection of the expression of signal molecules such as NLRP3, caspase-1, IL-1*β*, and IL-18; however, these studies are at the ex vivo level. Other studies have identified Gasdermin D (GSDMD) protein to be also involved in pyroptosis, with the exact mechanism still being undiscovered. These studies used chemotherapy drugs to activate caspase-3, which led to the cleavage of GSDME and subsequent pyroptosis. However, it was believed that caspase-3 led to apoptosis, which began the process of pyroptosis, but not direct involvement of caspase-3 in the pyroptosis. [42]. There is still much controversy about the role of pyroptosis in the CNS disease; that is, whether pyroptosis is beneficial or harmful in the disease and causing process is yet to be determined. Different diseases may have different outcomes, with the same disease having different effects at different stages of its development. In addition, whether pyroptosis has a role in tumor diseases [43, 44] is yet to be ascertained. Although the role of pyroptosis in different diseases and its signal pathways is still not very clear at present, with the gradual acceptance of pyroptosis by people, study on its specific effects and molecular mechanisms will surely bring new therapeutic methods and therapeutic targets for the treatment of diseases.

## 5. Conclusions

In summary, AC-YVAD-CMK, a well-known caspase-1 inhibitor, reduces brain damage caused by ICH in mice. This protective effect could be as a result of the inhibition of inflammatory signaling activation, attenuation of pyroptosis, and influence of microglial polarization. Preventing cleavage of caspase-1 into activated form by Ac-YVAD-CMK could, therefore, be a new way to prevent or attenuate inflammation and neurological injury after ICH.

## Data Availability

The data used to support the findings of this study are available from the corresponding author upon request.
